# Mutational analyses of novel rat models with targeted modifications in inflammatory bowel disease susceptibility genes

**DOI:** 10.1007/s00335-021-09868-2

**Published:** 2021-04-11

**Authors:** Hongsheng Men, Miriam A. Hankins, Anagha S. Bock, Benjamin P. Beaton, Daniel J. Davis, Kari L. Chesney, Elizabeth C. Bryda

**Affiliations:** 1 Rat Resource and Research Center, University of Missouri, Columbia, MO 65201 USA; 2 Department of Veterinary Pathobiology, University of Missouri, Columbia, MO 65201 USA; 3 Division of Animal Sciences, University of Missouri, Columbia, MO 65201 USA; 4 Present Address: Genus Plc., De Forest, WI 53532 USA; 5 Animal Modeling Core, University of Missouri, Columbia, MO 65201 USA; 6 Comparative Medicine Program, University of Missouri, Columbia, MO 65201 USA

## Abstract

Mutations and single base pair polymorphisms in various genes have been associated with increased susceptibility to inflammatory bowel disease (IBD). We have created a series of rat strains carrying targeted genetic alterations within three IBD susceptibility genes: *Nod2*, *Atg16l1*, and *Il23r,* using CRISPR/Cas9 genome editing technology. Knock-out alleles and alleles with known human susceptibility polymorphisms were generated on three different genetic backgrounds: Fischer, Lewis and Sprague Dawley. The availability of these rat models will contribute to our understanding of the basic biological roles of these three genes as well as provide new potential IBD animal models.

**Supplementary Information:**

The online version contains supplementary material available at 10.1007/s00335-021-09868-2.

## Introduction

Inflammatory bowel disease (IBD) refers to a group of diseases involving chronic inflammation of the gastrointestinal tract (GI). The main forms of IBD are Crohn’s disease (CD) and ulcerative colitis (UC). CD can affect any part of the GI, and affect extra-intestinal organ and tissue systems (Xavier and Podolsky 2007). UC, by comparison, is restricted to the colon and rectum, commonly manifests and spreads from a focal region, and involves only the mucosal layer of the GI. Both diseases have the potential to wreak havoc upon the GI system, cause life-threatening perforation and stricture of the gut, and result in life-long physical and emotional turmoil.

A high incidence of IBD is noted in Westernized societies with more than 1.5 million Americans and 2.5 million Europeans suffering from the disorder (Ananthakrishnan 2015; Graham and Xavier 2013; Rocchi et al. 2012). IBD is a complex disease in which both genetic and environmental factors play a role. Over 200 IBD-associated genes/loci have been identified (de Lange et al. 2017; Liu et al. 2015). Many of these loci are involved in immune homeostasis, inflammation, and autophagy. Other variants can lead to inappropriate responses to commensal bacteria resulting in IBD (Graham and Xavier 2013). However, genetic susceptibility alone is not sufficient for initiation and/or progression of disease, and growing evidence suggests a role for the diversity and composition of the gut microbiota as a factor in disease (Knights et al. 2014; Metwaly 2019; Sidiq et al. 2016).

Nucleotide-binding oligomerization domain-containing protein 2 (*NOD2*) plays an essential role in the immune system by controlling commensal bacterial flora in the intestine through its activity in intestinal epithelial cells, myeloid cells, and Paneth cells of the ileum (Lala et al. 2003; Ogura et al. 2003; Petnicki-Ocwieja et al. 2009). Three variants of *NOD2*; R702W, G908R, and the frameshift variant 1007 fs; are found with significantly higher frequency in CD patients than healthy individuals (Lesage et al. 2002). These variants result in signaling defects and reduction of GI intraluminal α-defensin as well as abnormal granulation of Paneth cells which leads to inflammatory lesions in the lining of the intestine (VanDussen et al. 2014; Wehkamp et al. 2005).

The Autophagy-related 16-like 1 (*ATG16L1*) gene functions in the process of macro-autophagy and is essential in the development of the double-membrane bound vesicle known as the autophagosome which is required to deliver unwanted cellular debris or intracellular bacteria to the lysosome for degradation (Glick et al. 2010; Levine and Kroemer 2008; Yang and Klionsky 2009). ATG16L1 is also involved in the exocytosis pathway of vesicles from the Paneth cells (Cadwell et al. 2008). A single base pair change from adenine to guanine in exon 9 of *ATG16L1* results in an amino acid change of threonine to alanine in the peptide sequence at position 300 (T300A). The T300A variant results in increased sensitivity to caspase-3 mediated cleavage of the ATG16L1 protein resulting in a reduction of overall ATG16L1 protein (Lassen and Xavier 2014; Murthy et al. 2014). During times of stress, the reduction in protein causes dysfunction and build-up of unwanted cellular debris and intracellular bacteria resulting in cellular necrosis and increased inflammation (Cadwell et al. 2010; Messer et al. 2013). This cellular inflammation is thought to be the inciting event for CD in patients carrying the T300A variant.

Rather than increasing susceptibility to CD, some interleukin-23 receptor (*IL23R*) variants have a protective role (Duerr et al. 2006; Sarin et al. 2011). A meta-analysis of 51 studies indicated that the *IL23R* R381Q variant decreased the risk of Crohn’s disease in individuals with higher mutation loads by 54% compared to those with lower mutational loads (Grigoras et al. 2015). Wild type IL23R is highly involved in the mediation of proinflammatory activities by producing IL-17 through activation of Th17 lymphocytes and through its interaction with IL-23, resulting in inflammation against infection (Naser et al. 2012; Vermeire et al. 2002). Genome-wide associated studies and deep resequencing have revealed multiple protective variants of *IL23Rα* against the development of CD and UC, including G149R, V362I, and R381Q (Beaudoin et al. 2013; Silverberg et al. 2009; Sivanesan et al. 2016). Protective coding variants of *IL23Rα* result in reduced cell surface expression of receptors and reduced IL-23 mediated signalling, thus reducing the inflammatory cascade potentiated by the wild type receptor (Sivanesan et al. 2016).

The goal of our study was to generate a collection of rat strains/stocks carrying genetic modifications in the *Nod2*, *Atg16l1* and *Il23r* genes. It is well known that rats offer several advantages in areas of behavioral, neurodevelopmental, and cardiovascular research; but to date, there are no rat models of IBD. The intestinal structure and the gut microbiome in humans are more similar to those in the rat than they are to those in the mouse (Homberg et al. 2017; Nagpal et al. 2018). The inflammatory response of Crohn’s disease is dominated by a T helper (Th) 1 immune response mechanism, which is a cell-mediated response characterized by an increased production of interferon (IFN)-γ (Fournié et al. 2001; Fuss et al. 1996; Strober and Fuss 2011). Similarly, Lewis rats are also prone to Th1 mediated organ-specific autoimmune diseases, such as experimental autoimmune uveoretinitis (Sakamoto et al. 2001). Therefore, based on these various similarities between humans and rats, we hypothesized that rats might be a better model species than mice to model IBD. We used CRISPR/Cas9 genome editing technology and three genetic backgrounds: Sprague Dawley (SD), Fischer 344 (F344), and Lewis (LEW) to generate new rat strains/stocks with genetic alterations in *Nod2*, *Atg16l1* and *Il23r*. These models represent important new animal resources for studying the biological functions of the genes as well as their role as susceptibility loci in the context of IBD.

## Materials and methods

### Animals

Four to five-week-old female rats were purchased from Envigo (Indianapolis, IN, USA) as zygote donors. They were housed in microisolator caging on ventilated racks in environmentally controlled rooms at 22 ºC under 10/14 h dark–light cycle (lights on 6:00 a.m.) with food and water ad libitum. This study was conducted in strict accordance with the recommendations in the *Guide for Animal Care and Use of Laboratory Animals* of the National Institutes of Health. The protocols for animal care and surgical procedures were approved by the Animal Care and Use Committee of the University of Missouri. The following rat strains have been assigned RRRC ID#s and are available from the Rat Resource and Research Center (Columbia, MO) at http://www.rrrc.us: F344-*Nod2*^*em5*^ (RRRC#914), F344-*Atg16l1*^*em8*^ (RRRC#896), and F344-*Il23r*^*em1*^ (RRRC#915). All other strains/stocks including LEW-*Nod2*^*em10*^ (RRRC#934), SD-*Nod2*^*em13*^ (RRRC#935), and SD-*Atg16l1*^*em2*^ (RRRC#897) have been cryopreserved and are also available upon request.

### Design and production of CRISPR reagents

Cas9 mRNA was purchased from MilliporeSigma (St Louis, MO, USA). Guide RNAs for knock-outs and knock-ins were designed using the DNA sequences as listed (Supplement Materials 2) and online design tool CRISPRdirect (http://crispr.dbcls.jp/) (Naito et al. 2015). Guide RNAs were ordered as gBlocks with a T7 promoter from Integrated DNA Technologies (IDT) Inc. (Coralville, Iowa, USA). Guide RNAs were generated by in vitro transcription using Ambion MEGAshortscript™ T7 Transcription Kit (Life Technologies, Carlsbad, CA, USA) and purified by the Ambion MEGAclear kit (Life Technologies) according to manufacturer’s protocols.

To create a rat model carrying the equivalent of the human *ATG16L1* rs2241880 (T300A) variant (ENST00000392017.9), a 200 bp single stranded oligonucleotide (ssODN) based on the rat Atg16l1-201 transcript (ENSRNOT00000024445.3) was used. This ssODN carries a cytosine to guanine change that results in a threonine to alanine substitution at position 300 of the protein (Fig. 3a, Supplement Materials 2). The human *IL23R Rs11209026* variant is a single nucleotide mutation leading to an arginine to glutamine change at position of 381 (R381Q) in the IL23R protein coded by transcript *IL23R-201* (ENST00000347310.10). In rats, the corresponding amino acid position is at 395 in the protein encoded by rat transcript *Il23r-201* (ENSRNOT00000010151.5). To create rats with the same mutation, a repair template in the form of a 200 bp ssODN using rat *Il23r* genomic sequence (Supplement Materials 2) with a base pair mutation corresponding to the human *IL23R Rs11209026* variant (Fig. 3b) was used. For both the T300A and R381Q knock-ins, a silent mutation was made to disrupt the PAM region adjacent to the gRNA target sequence in both ssODNs to prevent re-cutting of the knock-in sequence (Supplement Materials 2). All ssODNs were ordered as ultramers® from IDT.

### Microinjection

Female rats were super-ovulated by intraperitoneal injection of 20 IU PG600 (Valley Vet Supply, Marysville, KS, USA), followed by 40 IU human chorionic gonadotropin (hCG) (Calbiochem, San Diego, CA, USA) 50 hour (h) later. Zygotes were collected 23–24 h after hCG from copulation plug positive females and then cultured in mR1ECM (Miyoshi et al. 1995) at 37 ºC, with 5% CO_2_, 5% O_2_ and maximal humidity. For knock-out rat production, injection mixtures consisting of two gRNAs at 50 ng/µl each and 100 ng/µl Cas9 mRNA were introduced into zygotes through cytoplasmic injection. For knock-in rat production, injection mixtures consisting of one gRNA at 50 ng/µl, 100 ng/µl Cas9 mRNA, 100 ng/µl ssODN were introduced into zygotes through pronuclear injection. Zygotes that survived the injection were then surgically transferred at 30 zygotes/rat into pseudo-pregnant SD females within 1 h after microinjection.

### Mutational analysis

Potential deletion mutations were identified using the Surveyor® assay (Integrated DNA Technologies, Coralville, IA, USA). DNA was extracted with DNeasy® Blood & Tissue Kit (Qiagen, Valencia, CA, USA) using tail snip biopsies from 2-week-old pups. Samples were processed according to the manufacturer’s protocol. For each gene, the genomic region flanking the CRISPR targeting site(s) was amplified by PCR using three sets of primers designed to generate small, medium or large amplicons (Supplement Materials 1). PCR products were then purified and reannealed to form heteroduplexes. After reannealing, the products were cleaved by Surveyor® nuclease according to the manufacturer’s protocol and the presence of insertions/deletions (indels) was analyzed by gel electrophoresis.

High resolution melt analysis and Sanger sequencing were used to detect rats with targeted insertion of the single base pair changes based on the human variants. Briefly, genomic DNAs were isolated from tail snips of two-week old pups using protocols as described in Surveyor® assay section. Primers were designed to amplify 50–80 bp amplicons. PCR was conducted to amplify the sequences of interest using Precision Melt Supermix (Bio-Rad Laboratories, Hercules, CA) and CFX96-Real-Time System (Bio-Rad). The cycling conditions were: one cycle of 95 °C for 2 min; 40 cycles of 95 °C for 10 s, 60 °C for 30 s (with plate read), and 72 °C for 30 s. Melting cycle was 95 °C for 30 s, 60 °C for 1 min, and 65–95 °C for 10 s each with plate read every 0.2 °C. The Bio-Rad Precision Melt Analysis Software (Bio-Rad) was used to analyze PCR amplicons.

Sequencing was performed by first amplifying DNA samples from genome edited animals using primers located outside the targeted deletion sequence. Then, the cloning of amplicons was performed using TOPO® TA for sequencing (ThermoFisher Scientific, Waltham, MA, USA) according to manufacturer’s protocol. The TOPO® cloning reaction was transformed into One Shot® Competent Cells and grown on LB plates containing 50 µg/ml kanamycin overnight at 37 °C. Kanamycin-resistant bacterial colonies were selected and plasmid DNA was isolated using the PureLink® Quick Plasmid Miniprep Kit. Plasmid DNA was sequenced using M13 forward and reverse primers from the TOPO TA kit. The nucleotide sequences were analyzed using FinchTV software (Geospiza, Inc., Seattle, WA, USA).

## Results

### Nod2 and Atg16l1 knock-out models

**Fig. 1:**
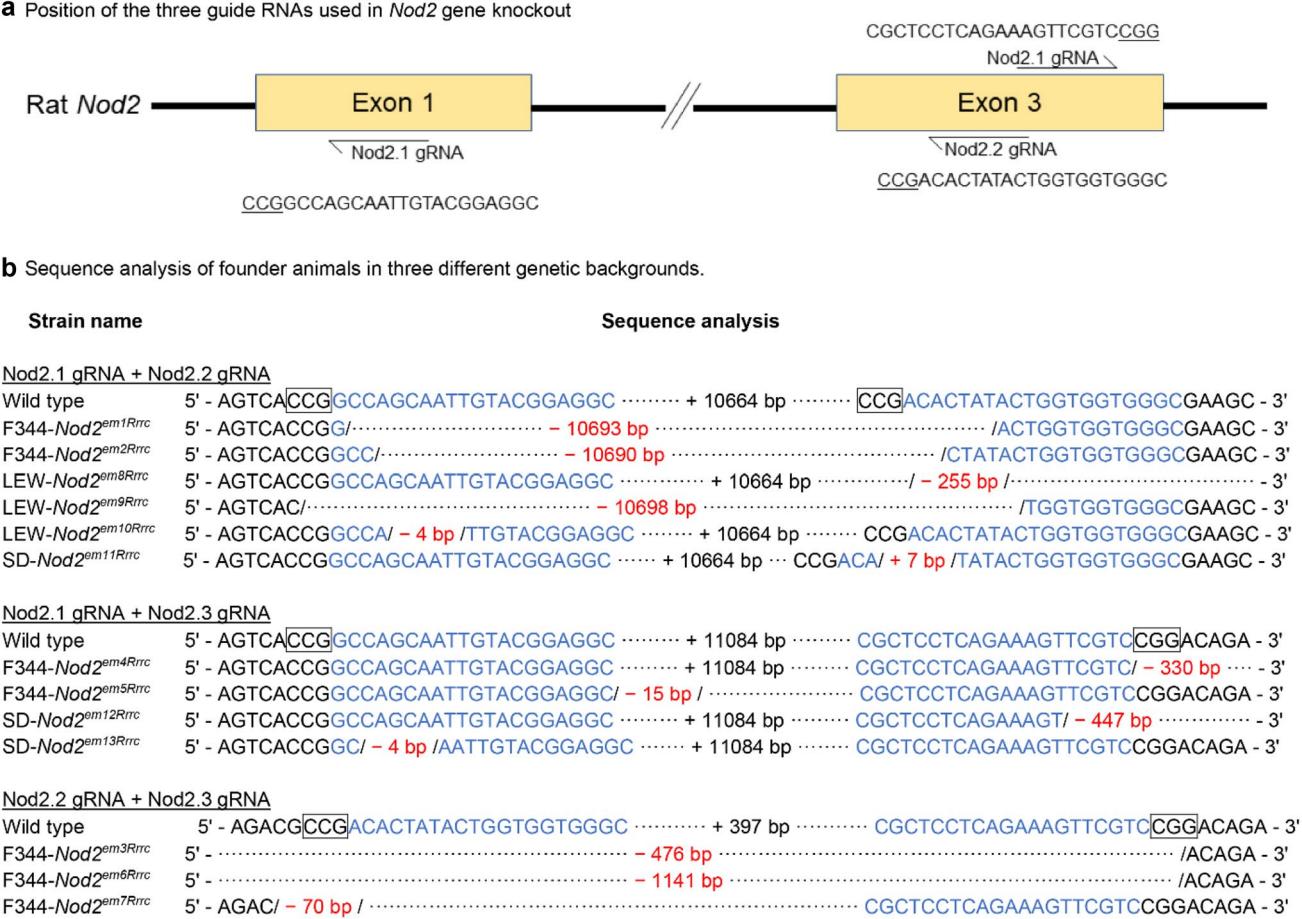
Mutational analysis of *Nod2* knock-out models. **a** Schematic illustration of strategy to knock out the rat *Nod2* gene using CRISPR/Cas9 genome editing. The positions of the guide RNAs are shown. The 3 nucleotides in the boxes are the PAM sequence. **b** Sequence analysis of founder animals with three genetic backgrounds. Wild type refers to NCBI reference sequence NC_005118.3. The nucleotides in blue are the gRNAs; the number after “ + ” sign in black indicates the number of nucleotides between gRNAs. The numbers after a “−“ sign in red indicate deletions

*Nod2* knock-out models were created in three genetic backgrounds: SD, F344 and LEW (Fig. 1). Resulting pups were initially screened by PCR genotyping using three sets of primers designed to the region targeted by each gRNA. The assays were designed to generate either small (40–60 bp), medium (~ 500 bp) or large (~ 1000 bp) amplicons (Supplement Materials 1). DNA from animals with evidence of a mutation based on this initial PCR screen, was further analyzed by nucleotide sequence analysis to characterize the exact nature of the mutation.

Animals that carried deletions in the *Nod2* gene were set up in breeding. We selected 7 founders from F344 (strain names: F344-*Nod2*^*em1−7Rrrc*^), three founders from SD (strain name: SD-*Nod2*^*em11−13Rrrc*^) and three founders from LEW (LEW-*Nod2*^*em8−10Rrrc*^) (Fig. 1). In all cases, germline transmission occurred, i.e., the deletion mutations were successfully transmitted from the original genetically manipulated animal to their offspring.

Genotyping results showed that using combinations of gRNAs resulted in mutations ranging from a few base pair deletions to over 10,000 base pair deletions in the *Nod2* gene. The overall deletion mutation efficiencies in the *Nod2* gene with different combinations of *Nod2* gRNAs are shown in Table 1. There were no strain-specific differences in overall mutation efficiencies among the three strains/stocks (*p* > 0.05). We also examined mutational efficiencies for a single combination of gRNAs (Nod2.1 and Nod2.2 gRNAs) in the three genetic backgrounds and again, no significant differences were observed among the three rat strains (Table 2, *p* > 0.05). However, there were significantly higher mutational rates in F344 rats resulting from injection of a combination of Nod2.1 + Nod2.2 gRNAs and a combination of Nod2.2 + Nod2.3 gRNAs compared with the those resulting from a combination of Nod2.1 and Nod2.3 gRNAs (Table 3, *p* < 0.05).

**Table 1:** Efficiencies of CRISPR-mediated *Nod 2* and *Atg16l1* knock-out, *Nod2* and *Il23r* knock-ins in rats from various strains/stocks

Gene	Strains	Mutation	Expected phenotype	# Zygotes transferred	# Pups produced (%^a^)	# Pups genotyped (%^b^)	# Pups with mutations (%^c^)
*Nod2*	SD	KO	CD susceptibility	95	24 (25)	24 (100)	16 (67)
*Nod2*	F344	KO	CD susceptibility	158	49 (31)	45 (92)	38 (84)
*Nod2*	LEW	KO	CD susceptibility	65	16 (25)	16 (100)	15 (94)
*Atg16l1*	SD	KO	CD susceptibility	226	67 (30)	54 (81)	30 (56)
*Atg16l1*	F344	T300A	CD susceptibility	120	22 (18)	14 (64)	1 (7)
*Il23r*	F344	R281Q	Protection from CD	261	97 (37)	89 (92)	2 (2)

*KO* knock-out, *CD* Crohn’s disease

^a^Ratio of pups produced to zygotes transferred

^b^Ratio of pups genotyped to pups produced

^c^Ratio of pups with mutation to pups genotyped

**Table 2:** Mutation efficiencies in various rat strains/stocks using a combination of Nod2.1 and Nod2.2 gRNAs

Strain	# Zygotes transferred	# Pups produced (%*)	# Pups genotyped (%**)	# Pups with mutations (%***)
SD	34	9 (27)	9 (100)	7 (78)^a^****
F344	60	11 (18)	11 (100)	11 (100)^a^
Lewis	65	16 (25)	16 (100)	15 (94)^a^

*Ratio of pups produced to zygotes transferred

**Ratio of pups genotyped to pups produced

***Ratio of pups with mutation to pups genotyped

****Same superscript letters indicate no statistical difference (*p* > 0.05, *χ*^2^ test)

**Table 3:** Mutagenesis efficiencies in F344 using different combinations of gRNAs targeting various exons of *Nod2* gene

gRNAs	# Zygotes transferred	# Pups produced (%*)	# Pups genotyped (%**)	# Pups with mutations (%***)
Nod2.1 + Nod2.2	60	11 (18)	11 (100)	11 (100)^a^****
Nod2.1 + Nod2.3	46	19 (41)	15 (79)	9 (60)^b^
Nod2.2 + Nod2.3	52	19 (37)	19 (100)	18 (95)^a^

*Ratio of pups produced to zygotes transferred

**Ratio of pups genotyped to pups produced

***Ratio of pups with mutation to pups genotyped

****Different superscript letters indicate statistical difference (*p* < 0.05, *χ*^2^ test)

**Fig. 2:**
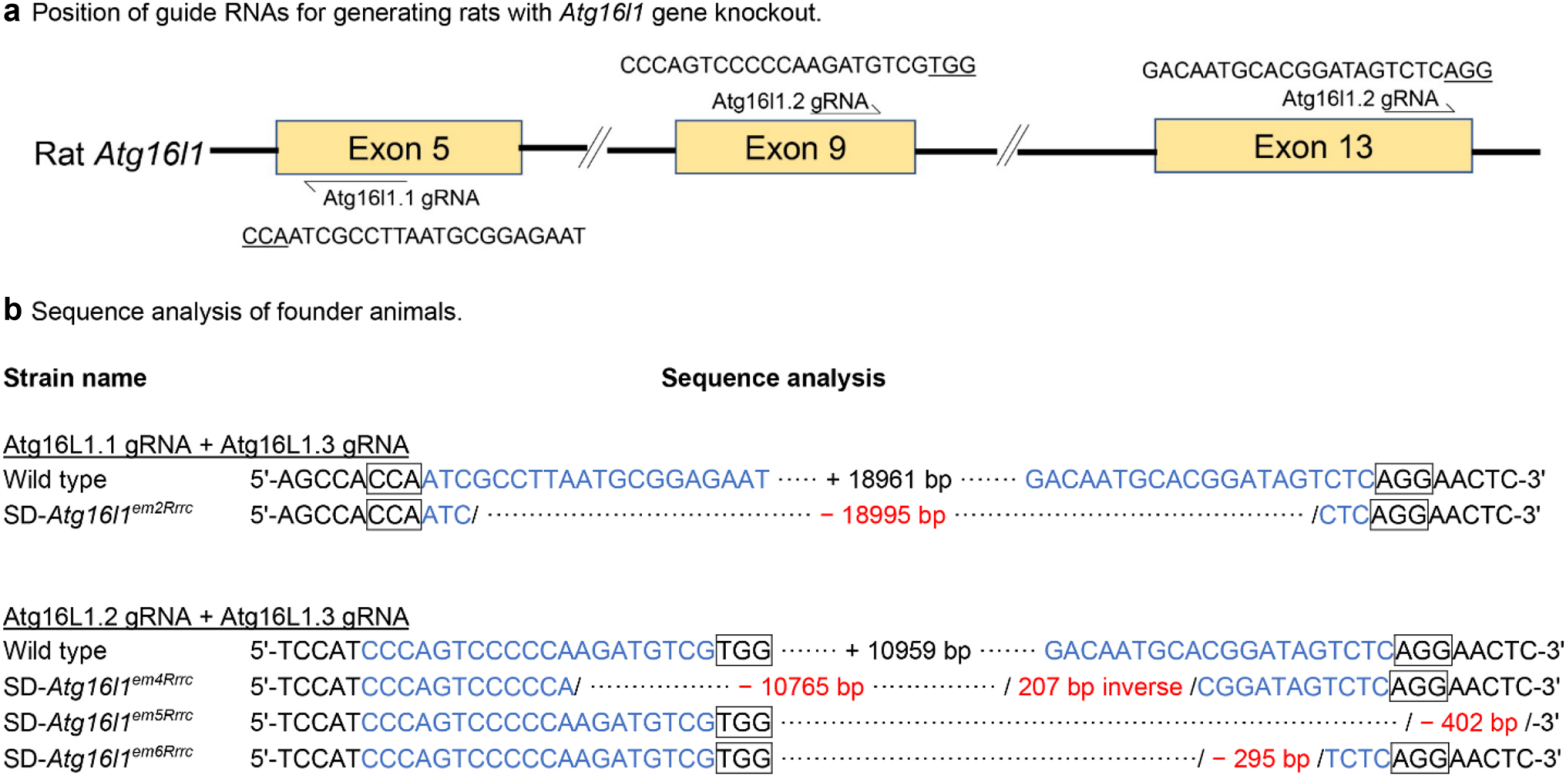
Mutation analysis of SD *Atg16l1* knock-out models. **a** Schematic illustration of knock-out strategy of rat *Atg16l1* gene using CRISPR/Cas9 genome editing. **b** Sequence analysis of founder animals. Wild type refers to NCBI reference sequence NM_001108809.3. The nucleotides in blue are the gRNAs; the number after “ + ” sign in black indicates the number of nucleotides between gRNAs. The numbers after a “−“ sign in red indicate deletions

The *Atg16l1* knock-out models were created on an SD genetic background (Fig. 2). The mutational efficiencies in SD rats are shown (Table 4). There were no significant differences in deletion mutation rates among the gRNA combinations. Mutant rats with deletions detected by PCR analysis were further analyzed by sequencing (Fig. 2). Four founder animals with deletion mutations in *Atg16l1* gene were kept and a stock name was given for each of the founder animals (SD-*Atg16l1*^*em2,4−6Rrrc*^).

**Table 4:** Mutagenesis efficiencies in Sprague Dawley using different combinations of gRNAs targeting various exons of the *Atg16l1* gene

gRNAs	# Zygotes transferred	# Pups produced (%*)	# Pups genotyped (%**)	# Pups with mutations (%***)
Atg16L1.1 + Atg16L1.2	33	9 (27)	9 (100)	6 (67)^a****^
Atg16L1.1 + Atg16L1.3	69	18 (26)	17 (94)	10 (59)^a^
Atg16L1.2 + Atg16L1.3	60	20 (33)	20 (100)	9 (45)^a^
Atg16L1.1 + Atg16L1.2 + Atg16L1.3	64	12 (19)	8 (67)	5 (63)^a^

*Ratio of pups produced to zygotes transferred

**Ratio of pups genotyped to pups produced

***Ratio of pups with mutation to pups genotyped

****Same superscript letters indicate no statistical difference (*p* > 0.05, *χ*^2^ test)

### Atg16l1 T300A and Il23r R381Q knock-in models

While we chose to generate the *Atg16l1* knock-outs on a SD background because this outbred stock is commonly used in rat studies and produces large numbers of embryos amenable to genetic manipulation, we switched to the F344 genetic background when making the human variant-specific strains. This was done to create variants using an inbred strain in order to limit background genetic variability.

**Fig. 3:**
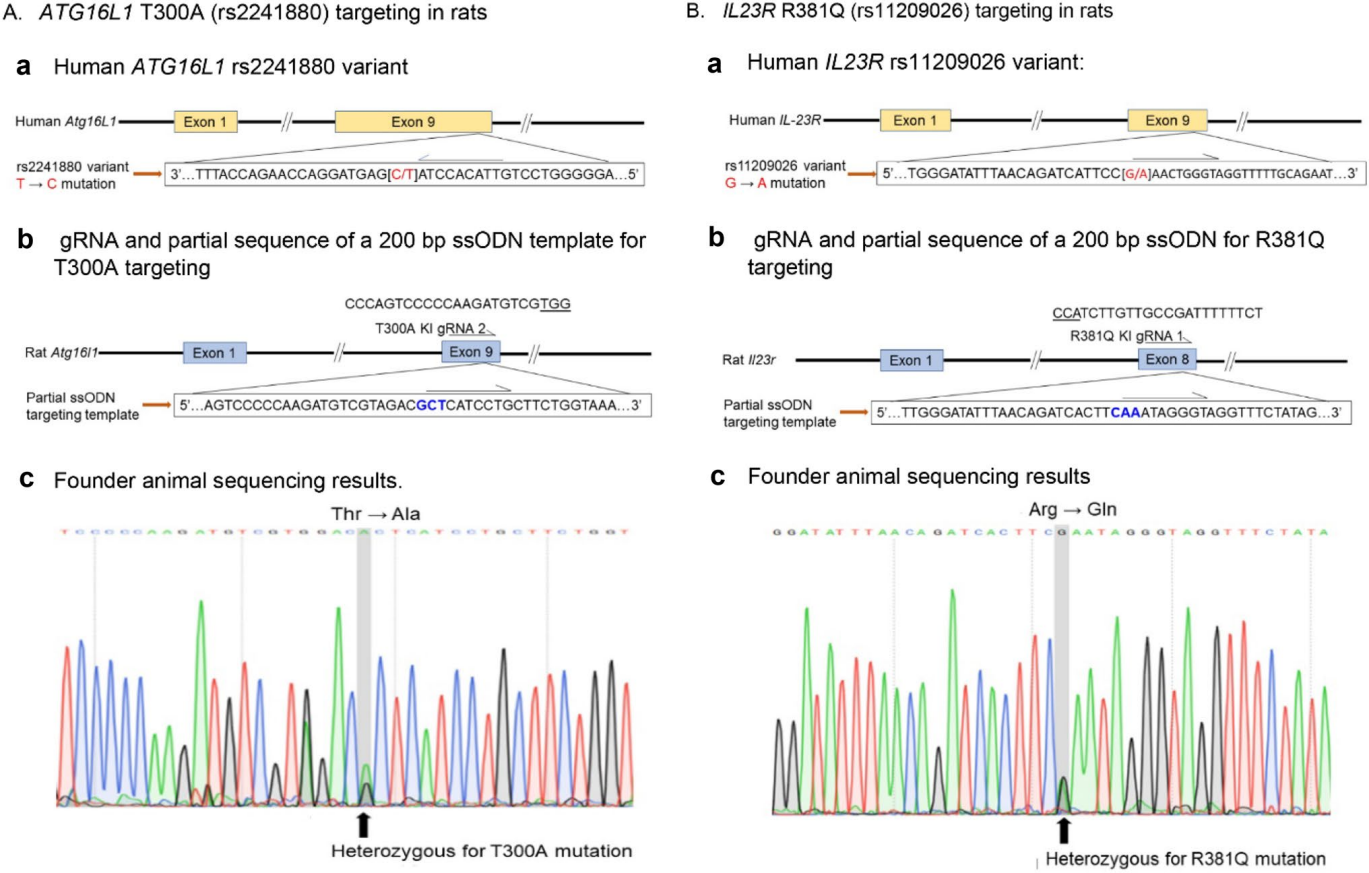
Creation of rat models carrying either *ATG16L1* T300A or *IL23R* R381Q mutations. **a** Targeting template design for generating rat models carrying human *Atg16L1* rs2241880 variant (T300A) using transcript *Atg16L1-203*. (a) Partial sequence of human *Atg16L1* T300A (rs2241880) variant showing the T → C mutation in the reverse sequence. (b) Partial sequence of ssODN targeting template for T300A rat generation. (c) Sequencing results showing heterozygous knock-in of T300A variant. **b** Targeting template design for generating rat models carrying *IL23R* rs11209026 variant using transcript *IL23R-201*. (a) Partial sequence of *IL23R* rs11209026 variant showing the G → A mutation in human *IL23R* gene. (b) Partial sequence of ssODN targeting template for *Il23r* rs11209026 variant rat generation. (c) Sequencing results showing heterozygous knock-in of *Il23r* R381Q variant

For the *Atg16l1* T300A knock-in model, a total of 120 zygotes injected with gRNA, Cas9 mRNA and a ssODN donor template carrying the mutation were surgically transferred into 4 recipients. Twenty-two pups were born but only 14 pups survived to 2 weeks of age, the time at which tissue is taken for genotyping. Of the 14 pups genotyped, 1 was confirmed to have the correct T300A mutation by high resolution melt and nucleotide sequence analysis (Fig. 3a). The knock-in efficiency (1 out of 14 animals genotyped) was 7%. The animal carrying the T300A variant was used to establish the strain which was designated F344-*Atg16l1*^*em8Rrrc*^.

For the *Il23r* R381Q knock-in model, a total of 261 injected zygotes were surgically transferred into 9 recipient dams and 97 live pups were born. Tissue was collected from a total of 89 2-week old pups. Two of the 89 pups were confirmed to have the correct R381Q mutation. This represents a KI efficiency of 0.8% out of 261 transferred zygotes and 2% out of 89 genotyped pups (Table 1). Only one of these animals was bred to establish the new strain (F344-*Il23r*^*em1Rrrc*^).

Initially, many of the animals carrying genetic alterations were backcrossed to wild type animals to confirm germline transmissibility of the mutation and dilute out any potential CRISPR/Cas9 off-target site alterations that might be present in the genetic background of the genetically manipulated animals. In all cases, we saw germline transmission. There was no evidence of changes in phenotype after repeated backcrossing as might occur if off-target mutations were segregating. Interestingly, we did note that for all of the independently generated strains involving deletions in the *Atg16l1* gene, we never recovered homozygous offspring suggesting that these mutations are embryonic lethal in the rat. Spermatozoa were cryopreserved for the majority of the strains/stocks but live colonies of F344-*Nod2*^*em5 Rrrc*^, F344-*Atg16l1*^*em8 Rrrc*^ and F344-*Il23r*^*em1 Rrrc*^ were established to allow more detailed characterization of the models. All strains/stocks are available from the RRRC (http://www.rrrc.us).

## Discussion

CRISPR/Cas9 genome editing has been demonstrated to be effective in inducing targeted mutations in diverse organisms including animals, plants and microbes (Bortesi et al. 2016). However, the efficiencies vary considerably in different species and/or across different laboratories. Many factors have been shown to contribute to the differences: the length of the single guide RNA (sgRNA), concentrations and forms (e.g. mRNA versus protein) of CRISPR/Cas9 reagents, genes being targeted as well as species (Dang et al. 2015; Hu et al. 2013; Li et al. 2013a, b; Ma et al. 2014). At the time our studies were initiated, little was known about the efficiency of generating knock-outs or knock-ins in the rat using CRISPR/Cas9 genome editing technology. In the course of creating new rat strains/stocks with targeted mutations in the IBD susceptibility gene *Nod2* using fixed concentrations of CRISPR reagents (Cas9 mRNAs and gRNAs), we had the opportunity to assess the overall deletion mutation efficiencies. We saw no genetic background-related differences in the mutation rate within the *Nod2* gene when comparing SD, F344 and LEW. However, we did note that different *Nod2*-targeting gRNAs resulted in differences in mutational efficiencies within the *Nod2* gene. This is in agreement with previous observations by others (Ran et al. 2013; Wang et al. 2013). Several factors may contribute to the gRNA’s efficacy in inducing mutations. The GC content of the gRNA as well as which DNA strand and gene exon are targeted have been associated with the different mutational efficiencies. For example, using gRNAs with very low or high GC content, targeting the transcribed DNA strand and editing the last exon of a gene are usually less effective (Bortesi et al. 2016; Wang et al. 2014). The gRNA’s secondary structure, which is determined by the sequence features of gRNAs, also affect the interaction between gRNA and the Cas9 proteins and consequently, influences the mutational efficiencies (Dang et al. 2015; Doench et al. 2014; Wang et al. 2014). The efficiencies of generating the desired knock-in variants based on the total number of pups genotyped were 7.1% and 2% for *Atg16l1* and *Il23r* respectively. Our knock-in efficiencies are in line with those previously reported using similar approaches in both mice and rats (Inui et al. 2014; Yoshimi et al. 2014).

An important consideration when using CRISPR/Cas9 and designing appropriate gRNAs is to ensure that there is protospacer adjacent motif (PAM) upstream of the desired genomic DNA target. The PAM is a 3 nucleotide base pair sequence that is recognized by Cas9 and is required for its function. It has been demonstrated that the efficiencies of incorporating point mutations can be affected by the distance between the site being targeted for point mutation and the PAM sequence (Inui et al. 2014). In our studies, the target nucleotide site for the *Atg16l1* T300A KI is 6 bases downstream of the PAM sequence and the point mutation target site for the *Il23r* E281Q variant is 49 bases downstream of the PAM sequence. The differences in these distances may have contributed to the different efficiencies in generating the two knock-in strains. However, it is equally possible that the differences are gene-specific differences related to the genomic architecture of the regions where the two genes are located.

In summary, we have shown that CRISPR/Cas9 genome editing is an effective method for generating genetically modified rat strains/stocks. We have described several novel rat lines carrying deletion mutations in the rat *Nod2* and *Atg16l1* genes as well as knock-in rat lines carrying point mutations representative of the human *ATG16L1* T300A variant and *IL23R* R381Q variants. These are the first rat models specifically generated for the study of IBD susceptibility. All genetic alterations were germline transmissible and these strains are now available to the scientific community through the RRRC, a repository and distribution center for important rat models used in research. Continued phenotypic characterization of the models is ongoing but preliminary data shows that they faithfully recapitulate human disease. Not only will these rat strains/stocks allow investigators to address basic questions related to the biological roles of *Nod2* and *Atg16l1* but they will contribute to understanding of IBD disease mechanisms, disease susceptibility and ultimately, to the development of therapeutic strategies for IBD.

## Supplementary Information

Below is the link to the electronic supplementary material.

Supplementary file1 (DOCX 306 kb)

## Data Availability

The models described in this manuscript are available from the Rat Resource and Research Center (www.rrrc.us).
