# Supplementary material for: Mutational analyses of novel rat models with targeted modifications in inflammatory bowel disease susceptibility genes
Source: Mamm Genome. 2021 Apr 11;32(3):173–82. doi: 10.1007/s00335-021-09868-2 (PMC8128796; doi:10.1007/s00335-021-09868-2)

| **Supplement material 1**  **Primers used for genotyping rat *Nod2* deletion mutations** | |  |
| --- | --- | --- |
| **Primers** | **5' to 3'** | **Amplicon size (bp)** |
| RNOD2.1GENO80F | CTTCATCCCACCAGAGACCTG | 80 |
| RNOD2.1GENO80R | TCCAATATGGCTTCTACATGGCTG |  |
| RNOD2.1GENO649F | ACAGCGCCTGACTACAACTG | 649 |
| RNOD2.1GENO649R | GAGTCACAGTCTTGGTGGCTA |  |
| RNOD2.1GENO1077F | GAAGTCCCACTGCTTGGGTT | 1077 |
| RNOD2.1GENO1077R | GGCGTTATTCCGTGGGACTG |  |
| RNOD2.2GENO40F | CTGAACAAAGACGCCGACAC | 40 |
| RNOD2.2GENO40R | CTTCGCCCACCACCAGTATAG |  |
| RNOD2.2GENO620F | TGGCCATATCTTTGCAGGGG | 620 |
| RNOD2.2GENO620R | GGCCAACAGCAATGCTCAAA |  |
| RNOD2.2GENO1307F | TGGCATGTGCCATCCTTGTT | 1307 |
| RNOD2.2GENO1307R | CTCAGAGTCCACCTGTGCTG |  |
| RNOD2.3GENO53F | TGTCAGCGCTCCTCAGAAAG | 53 |
| RNOD2.3GENO53R | GAAGCCCTTAAGGTGGCACT |  |
| RNOD2.3GENO482F | ATGCCTGCAAGGTACTGACC | 482 |
| RNOD2.3GENO482R | CTCAGAGTCCACCTGTGCTG |  |
| RNOD2.3GENO1401F | AAGTTCCGGTTCACAGACCG | 1401 |
| RNOD2.3GENO1401R | ACTCCTGGATCCCAACAGTG |  |
|  |  |  |
| **Primers used for genotyping rat *Atg16l1* deletion mutations** | |  |
| **Primers** | **5' to 3'** | **Amplicon size (bp)** |
| RAtg16l1.1GENO44F | GATGGCTGAGAAGGCCCAAG | 44 |
| RAtg16l1.1GENO44R | TCTCCGCATTAAGGCGATTG |  |
| RAtg16l1.1GENO419F | CAGGTGCCTGGCTGTGTTAC | 419 |
| RAtg16l1.1GENO419R | TCTACCACCTACCACCCTGAC |  |
| RAtg16l1.1GENO1146F | TGCTTGCTCAAGCTCACCTA | 1146 |
| RAtg16l1.1GENO1146R | ACTCCTGCATGTCCTGTCAC |  |
| RAtg16l1.2GENO55F | CGCTCTGTCTCTTCCATCCC | 55 |
| RAtg16l1.2GENO55R | AAGCAGGATGAGTGTCCACG |  |
| RAtg16l1.2GENO527F | ACTGCTTCAGTGAGGGCTTT | 527 |
| RAtg16l1.2GENO527R | TCCCTAACAAGTTCTGTGGGC |  |
| RAtg16l1.2GENO1127F | AACCGTGTTTCAGAGTGCCA | 1127 |
| RAtg16l1.2GENO1127R | AGGCTCTTAGTGGTGGGAGT |  |
| RAtg16l1.3GENO47F | TCTCTGCCAAGTTCCTGCTG | 47 |
| RAtg16l1.3GENO47R | ACTTCCTGAGACTATCCGTGC |  |
| RAtg16l1.3GENO590F | CCACAGCGGGAAAGTTCTCT | 590 |
| RAtg16l1.3GENO590R | GCTGGGCCCACAAGACTATT |  |
| RAtg16l1.3GENO1087F | GTTGCTTTGCCCTGTGATGG | 1087 |
| RAtg16l1.3GENO1087R | GCTGGGCCCACAAGACTATT |  |


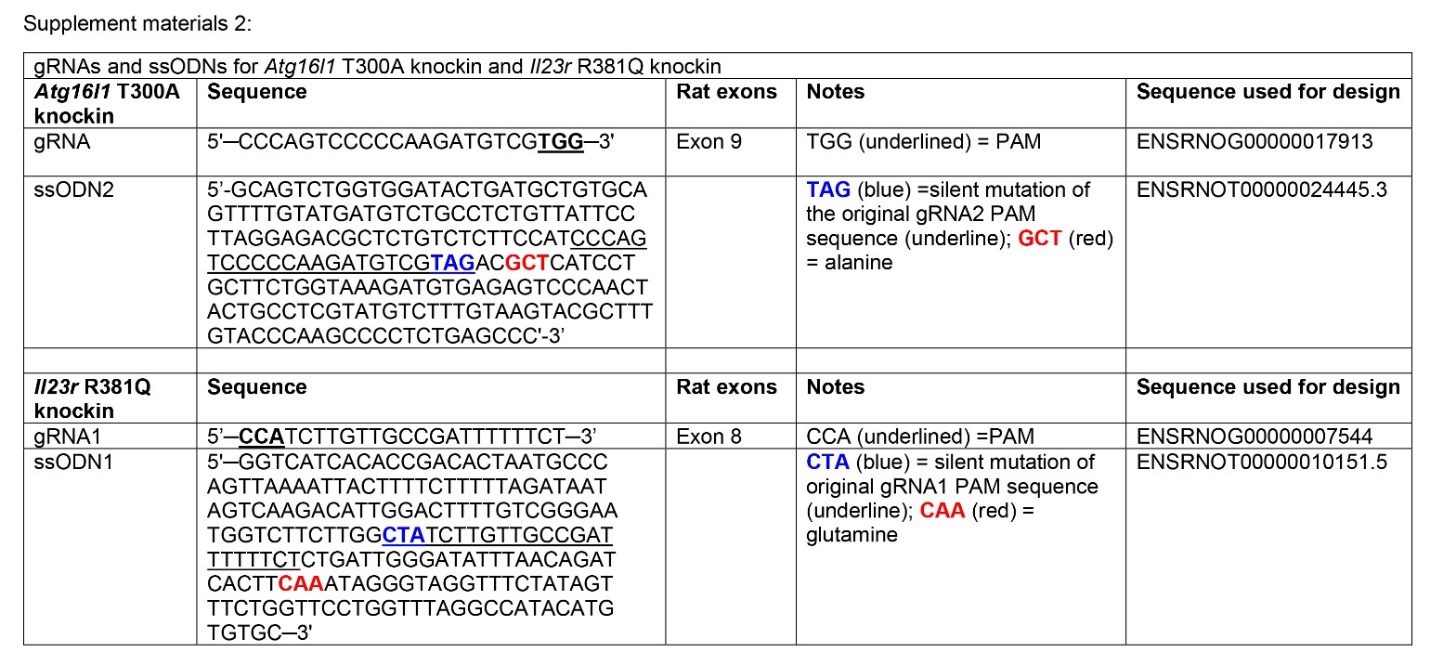

Supplement: Supplementary file 1 — Supplementary file1 (DOCX 306 kb) [file 335_2021_9868_MOESM1_ESM.docx]
